# Hepatoblastoma: glutamine depletion hinders cell viability in the embryonal subtype but high *GLUL* expression is associated with better overall survival

**DOI:** 10.1007/s00432-021-03713-4

**Published:** 2021-07-07

**Authors:** Andreas Schmidt, Angela Armento, Ovidio Bussolati, Martina Chiu, Verena Ellerkamp, Marcus O. Scharpf, Philip Sander, Evi Schmid, Steven W. Warmann, Jörg Fuchs

**Affiliations:** 1 Department of Paediatric Surgery and Paediatric Urology, University Children’s Hospital, Eberhard Karls University Tuebingen, Hoppe-Seyler-Strasse 3, 72076 Tuebingen, Germany; 2 Department for Ophthalmology, Institute for Ophthalmic Research, University of Tuebingen, Elfriede-Aulhorn-Straße 7, 72076 Tuebingen, Germany; 3 Department of Medicine and Surgery (DiMeC), University of Parma, Parma, Italy; 4 Institute for Pathology and Neuropathology, Department of General Pathology and Pathological Anatomy, Eberhard Karls University Tuebingen, Liebermeisterstr. 8, 72076 Tuebingen, Germany

**Keywords:** Hepatoblastoma, Hepatocellular carcinoma, Glutamine depletion, Glutamine synthetase, Asparagine synthetase, Asparaginase

## Abstract

**Purpose:**

Glutamine plays an important role in cell viability and growth of various tumors. For the fetal subtype of hepatoblastoma, growth inhibition through glutamine depletion was shown. We studied glutamine depletion in embryonal cell lines of hepatoblastoma carrying different mutations. Since asparagine synthetase was identified as a prognostic factor and potential therapeutic target in adult hepatocellular carcinoma, we investigated the expression of its gene *ASNS* and of the gene *GLUL,* encoding for glutamine synthetase, in hepatoblastoma specimens and cell lines and investigated the correlation with overall survival.

**Methods:**

We correlated *GLUL* and *ASNS* expression with overall survival using publicly available microarray and clinical data. We examined *GLUL* and *ASNS* expression by RT-qPCR and by Western blot analysis in the embryonal cell lines Huh-6 and HepT1, and in five hepatoblastoma specimens. In the same cell lines, we investigated the effects of glutamine depletion. Hepatoblastoma biopsies were examined for histology and *CTNNB1* mutations.

**Results:**

High *GLUL* expression was associated with a higher median survival time. Independent of mutations and histology, hepatoblastoma samples showed strong *GLUL* expression and glutamine synthesis. Glutamine depletion resulted in the inhibition of proliferation and of cell viability in both embryonal hepatoblastoma cell lines. *ASNS* expression did not correlate with overall survival.

**Conclusion:**

Growth inhibition resulting from glutamine depletion, as described for the hepatoblastoma fetal subtype, is also detected in established embryonal hepatoblastoma cell lines carrying different mutations. At variance with adult hepatocellular carcinoma, in hepatoblastoma asparagine synthetase has no prognostic significance.

## Introduction

The amino acid glutamine (Gln) is involved in key cellular processes, such as energy metabolism, nucleotide biosynthesis, the balance of redox potential, and in various signaling pathways (DeBerardinis and Cheng 2010). Dependence on Gln has been described for several tumors, and Gln metabolism has been identified as a promising target in tumor therapy (Lukey et al. 2013; Martinez-Outschoorn et al. 2017; Still and Yuneva 2017). Therapeutic options that target Gln synthesis and metabolism are being investigated for selected tumors (Mates et al. 2020; Schulte, 2018; Ye, 2018).

Gln is synthesized by the enzyme glutamine synthetase (GS or GLUL glutamate-ammonia ligase), encoded by the gene *GLUL*. In hepatic models, the transcription of this gene is primarily regulated by the Wnt/β-catenin pathway. Activation of this signaling pathway through Wnt ligands prevents β-catenin degradation in the cytosol, allowing β-catenin to migrate into the nucleus where it increases the transcription of *GLUL* by interacting with the transcription factors TCF and LEF (Monga 2015; Willert and Nusse 1998). Mutations in the gene for β-catenin, *CTNNB1*, result in reduced degradation and, thus, in increased *GLUL* expression and activity (Cadoret, 2002; Lopez-Terrada, 2009b).

Hepatoblastomas (HB), the most common malignant liver tumors in children, carry up to 90% deletions and/or missense mutations in the *CTNNB1* gene (Cairo, 2008; Eichenmuller, 2014; Koch et al. 1999; Lopez-Terrada et al. 2009b; Sumazin, 2017). Activation of the Wnt/β-catenin pathway is reported in 97% of hepatoblastomas, given that mutations in other components of the pathway may also lead to its activation (Jia, 2014; Koch et al. 1999, 2004; Sumazin et al. 2017).

Histology of HB presents heterogeneous tumors consisting mainly of epithelial components with fetal and embryonal parts, as well as mesenchymal and undifferentiated small cell components (Lopez-Terrada, 2014). Prognosis correlates with histology and ranges from very good, for the pure fetal type, to poor for the undifferentiated small cell type (Czauderna et al. 2014; Haas et al. 2001, 1989; Malogolowkin et al. 2011; Zimmermann 2005).

Studies on the effect of glutamine depletion were performed with the hepatoblastoma cell line HepG2, representing the fetal tumor subtype (Crippa, 2017; Lopez-Terrada et al. 2009a) and on its murine xenografts. Glutamine depletion reduced cell viability in vitro and the growth of xenografts in vivo (Chiu, 2014; Tardito, 2011).

Since the tumor subtypes are different in gene expression and metabolism, we investigated the effect of glutamine depletion, *GLUL* expression, and GS abundance in the hepatoblastoma cell lines Huh-6 and HepT1, which differ in their mutations and, in contrast to the fetal-type HepG2, derive from tumors of the embryonal subtype (Crippa et al. 2017; Koch et al. 1999; Pietsch et al. 1996).

Because of the relationship between Gln and asparagine (Asn) metabolism, we investigated expression of the gene *ASNS,* which encodes asparagine synthetase (ASNS), and ASNS abundance along with *GLUL* expression and GS abundance in the two cell lines and in biopsies of hepatoblastoma patients. Besides histological staining, we examined patient biopsies for *CTNNB1* mutations. Additionally, since ASNS was identified as a prognostic parameter in hepatocellular carcinoma (Zhang, 2013), we investigated the association between overall survival of patients and *GLUL* and *ASNS* expression, exploiting publicly available microarray data.

## Materials and methods

### Patients and tissue samples

Expression of *GLUL* and *ASNS* was evaluated from published expression databases (http://www.ebi.ac.uk/microarray-as/ae/; E-MEXP-1851 (Cairo et al, 2008)). The databases include 25 HB samples and 4 non-tumor liver samples, which were analyzed by Affymetrix oligonucleotide array (Affymetrix U133A2.0; Affymetrix, Santa Clara, CA). Relative expression of the genes is given as signal log ratio (SLR). For all 25 HB samples, the differentiation into the molecular subclasses C1- and C2-type, as well as survival data, has been previously reported (Cairo et al. 2008). For one patient follow-up data are missing, and the relevant data were censored in the analysis. We used these published microarray and clinical data to analyze the correlation between gene expression and overall survival.

Five samples from our human hepatoblastoma tissue collection with epithelial-fetal (*n* = 3) epithelial-embryonal (*n* = 1) and mixed epithelial-mesenchymal (*n* = 1) histology were analyzed for mutations on exon 3 of *CTNNB1* as well as for *GLUL* and *ASNS* expression on mRNA level and abundance of the respective proteins.

### Western Blot

Hepatoblastoma tumor specimens were immediately fixed in liquid nitrogen and stored at − 80 °C for further analysis Hepatoblastoma tissue samples were homogenized with an Ultra Turrax (IKA) instrument in RIPA lysis buffer (Cell Signaling, Germany) containing a cocktail of protease inhibitors (Roche) and centrifuged at 14,000 g for 20 min at 4 °C. Aliquots of 30 µg of proteins in Roti Load^®^ buffer 4x (Carl Roth) were warmed at 95 °C for 5 min and loaded on a 10% gel for SDS-PAGE.

Cells were lysed in a buffer containing 20 mM Tris–HCl, pH 7.5, 150 mM NaCl, 1 mM EDTA, 1 mM EGTA, 1% Triton, 2.5 mM sodium pyrophosphate, 1 mM β-glycerophosphate, 1 mM Na_3_VO_4_, 1 mM NaF, 2 mM imidazole and a cocktail of protease inhibitors (Protease Inhibitor Cocktail, Sigma-Aldrich). Lysates were transferred in Eppendorf tubes, sonicated for 5 s and centrifuged at 12,000 g for 10 min at 4 °C. Protein determination was performed with Coomassie Brilliant Blue G-250 acid solution (Bio-Rad protein assay). Absorbance at 750 nm was read, and protein content was calculated from bovine serum albumin standards. Aliquots of 30 μg of proteins were mixed with Laemmli buffer 4x (250 mM Tris–HCl, pH 6.8, 8% SDS, 40% glycerol, and 0.4 M DTT), warmed at 95 °C for 5 min and loaded on a 10% gel for SDS-PAGE.

After electrophoresis, proteins were transferred to nitrocellulose membranes (Millipore). To block non-specific binding sites, an incubation of 1 h at room temperature in 10% non-fat dried milk (Carl Roth, Germany) in TBS-T solution was performed. The next step was the incubation of the blots at 4 °C overnight with the following antibodies diluted in a 5% BSA TBS-Tween solution: anti-GS (rabbit, polyclonal, 1:1000, GeneTex, #GTX109121), anti-ASNS (rabbit, monoclonal, 1:500, Cell Signaling, Frankfurt am Main, Germany, #92479) and anti-GADPH (rabbit, monoclonal, 1:2000, Cell Signaling, #2118). Blots were then washed and exposed for 1 h at room temperature to HRP-linked anti-rabbit antibody (Cell Signaling, #7074) diluted 1:3000 in 5% non-fat dried milk. Visualization of immunoreactivity was performed with a Western Sure Premium Chemiluminescent Substrate (LI-COR, Lincoln, Nebraska, USA). Specific bands were quantified by LI-COR Image Studio software (LI-COR, Lincoln, Nebraska, USA). Levels of each protein were expressed as the ratio of signal intensity for the target protein relative to that of GAPDH (Schmidt et al. 2014).

### Cell culture

The two HB cell lines Huh-6 (Doi 1976) and HepT1 (Pietsch et al. 1996) were used for this study. The Huh-6 cell line was bought from the Japanese Collection of Research Bioresources (Huh-6 Clone 5; JCRB0401, RRID:CVCL_1296). Members of our research laboratory were part of the group that implemented the hepatoblastoma cell line HepT1 (RRID:CVCL_G003). As a result, we have access to this cell line. All used cells were tested negative for mycoplasma. Mycoplasma detection was determined according to the manufacturer’s instructions (MycoAlert™, Mycoplasma Detection Kit, Lonza, Basel, Switzerland). Cells were grown in high-glucose (4.5 g/l) Dulbecco’s modified Eagle’s medium (DMEM); supplemented with 2 mM glutamine, 10% of fetal bovine serum (FBS), 100 U/ml penicillin, and 100 µg/ml streptomycin. All reagents were from Biochrom, Berlin, Germany. Cells were seeded at a density of 2 × 10^4^ cells per well in complete growth medium in 24-well plates in triplicates.

For viability assays, after 24 h the culture medium was substituted with fresh complete medium supplemented with asparaginase (ASNase) (*E. chrisanthemy* ASNase, Erwinase®, Jazz Pharmaceuticals, UK), the GS inhibitor methionine-L-sulfoximine (MSO) (Sigma-Aldrich, Munich, Germany), or a combination of both substances. ASNase was used at seven concentrations from 0.003 to 10 U/ml for cell number experiments, and at six concentrations from 0.003 to 1 U/ml for MTT-assay, while MSO was used at 1 mM. The effects of ASNase and MSO on cell cultures were assessed counting viable cells with a Coulter Z1 particle counter 72 h later or by means of a colorimetric MTT-assay measuring the reduction of tetrazolium salts to formazan derivatives by functional mitochondria. Lysis buffer (DMSO, SDS, acid) were added to solubilize the blue MTT-formazan product. The assays were performed in triplicates as originally described (Mosmann 1983). Absorbance was measured at 570 nm.

To determine the effect of ASNase on mRNA and protein levels of GS and ASNS, after 24 h the culture medium was substituted with fresh complete medium supplemented with ASNase (1 U/ml), ASNase (1 U/ml) and MSO (1 mM), or with standard growth medium for controls. mRNA and protein were analyzed 6 and 24 h later, and protein also after 48 h.

### RT-qPCR analysis

Total RNA was isolated from Huh-6 and HepT1 cells using the GenElute™ Mammalian Total RNA Miniprep Kit (Sigma-Aldrich, Munich, Germany). Hepatoblastoma samples were immediately preserved in RNAlater^©^ (Qiagen, Hilden, Germany) and stored at − 80 °C for further RNA analysis. The tissue samples were homogenized in RLT buffer (including DTT) using an Ultra Turrax (IKA, Staufen, Germany) instrument. Total RNA was isolated from hepatoblastoma samples using the RNeasy mini kit by following the manufacturer's instructions (Qiagen, Hilden, Germany). cDNA synthesis was performed using High capacity cDNA Reverse Transcription Kit (Applied Biosystems, Waltham, Massachusetts, USA). Reverse transcription and semi-quantitative PCR were performed as described by Chiu et al, 2014. Primers used for *ASNS* were 5’ GATTGCCTTCTGTTCAGTGTCT 3’ (for) and 5’ GGGTCAACTACCGCCAACC 3’ (rev), for *GLUL* 5’ TCATCTTGCATCGTGTGTGTG 3’ (for) and 5’ CTTCAGACCATTCTCCTCCGG 3’ (rev), and for *Axin-2* 5' AGGGAGAAATGCGTGGATAC 3' (for) and 5' TGGAATCAATCTGCTGCTTC 3' (rev). Housekeeping genes were Ribosomal protein L15 (*RPL15*) 5’ GCAGCCATCAGGTAAGCCAAG 3’ (for) and 5’ AGCGGACCCTCAGAAGAA AGC 3’ (rev) for the experiments with cells and *TBP* (TATA binding protein, 5’ GCC CGA AAC GCC GAA TAT 3’ (for) and 5’CCG TGG TTC GTG GCT CTC 3’ (rev)) for experiments with tissue samples. TBP (TATA Box Binding Protein) was used as reference gene in RT-PCR analysis as it is directly involved in transcriptional activity of a gene and correlates with the transcriptional activity of the promoter region. GAPDH, on the other hand, is one of the top 20 housekeeper with the highest and most consistent average expression (She et al. 2009) and was thus chosen for comparison of protein expression of respective genes**.** For data analysis, the ∆∆CT method was used (Livak and Schmittgen 2001). Specificity of PCR products was confirmed by analysis of a melting curve. Real-time amplifications were performed on a C1000™ Thermo Cycler (Bio-rad, Feldkirchen, Germany).

### Next-generation-sequencing (NGS) of *CTNNB1* exon 3

Genomic DNA was extracted from macro-dissected 5 µm paraffin sections using the Maxwell^®^ RSC DNA FFPE Kit and the Maxwell^®^ RSC Instrument (Promega, Madison, WI, USA) according to the manufacturer’s instructions.

*CTNNB1* exon 3 was investigated by next generation sequencing with a single amplicon using the Ion Amplicon Library Preparation Fusion Method (ThermoFisher Scientific, Waltham, MA, USA) according to the manufacturer’s protocol (gene specific primer sequences identical to Sanger sequencing: Fwd: 5’-TGGAACCAGACAGAAAAGCG-3’ and Rev: 5’-CAGGTACCGTGCGACATC-3’). Amplicons were purified and quantified applying Agencourt AMPure XP magnetic beads (Beckman Coulter, Brea, CA, USA) and the Qubit dsDNA HS Assay Kit (Thermo Fisher Scientific), respectively. Amplicons were diluted to 5 pM each and pooled. Clonal amplification and semiconductor sequencing was done according to the manufacturer’s manuals using the Ion 510™ and Ion 520™ and Ion 530™ Kit—Chef (ThermoFisher Scientific) on the Ion Chef™ Instrument (ThermoFisher Scientific) and the Ion 520™ Chip Kit on the Ion GeneStudio™ S5 Prime system (ThermoFisher Scientific). BAM Files were generated with Torrent Suite 5.10.2. Sequences were visualized and evaluated using the freely available software Integrative Genomics Viewer (IGV, Broad Institute).

### Statistical analysis

Survival curves were calculated using the Kaplan–Meier method and compared using the log-rank test. Data of *GLUL* and *ASNS* expression levels were expressed as mean ± SD. The relationship between *GLUL* and *ASNS* expression was analyzed with the use of Pearson correlation. Two-tail t test for unpaired samples was used to detect differences between groups. *p* values below 0.05 were considered statistically significant. All statistical analyses were conducted with the use of GraphPad Prism 5.0™.

## Results

### Expression of *GLUL* and *ASNS* in hepatoblastoma

**Fig. 1:**
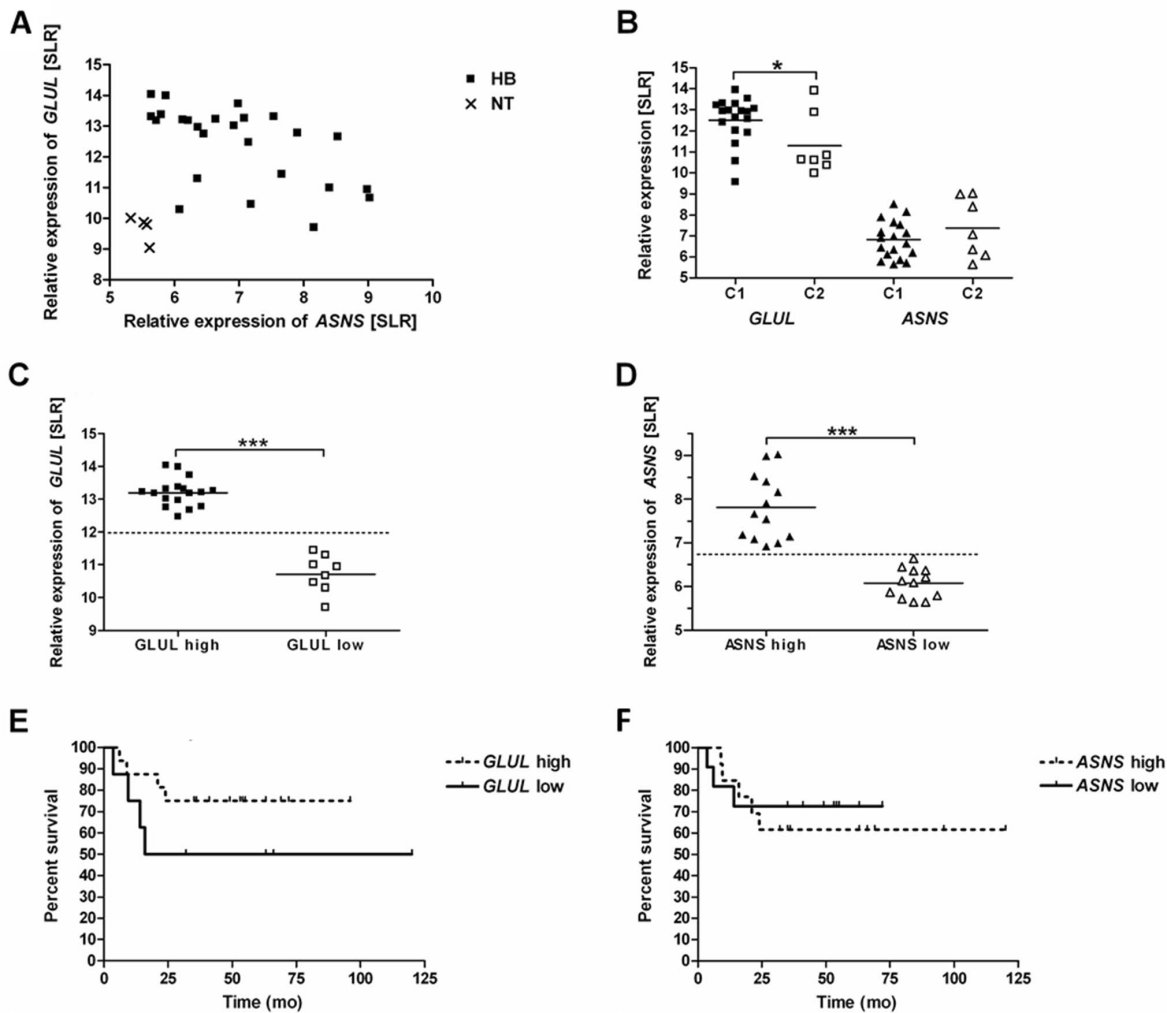
*GLUL* and *ASNS* expression in HB and overall survival. *GLUL* and *ASNS* expression levels of HB (*n* = 25) and normal liver tissues (NT) (*n* = 4) samples are shown. Each data point reflects the *GLUL* and *ASNS* level of one sample. Relative expression levels of genes are given as signal log ratio (SLR). **A** Scatter plot of the data. Expression of *GLUL* and *ASNS* is significant higher in HB than in NT (*p* = 0.0003 and *p* = 0.01, respectively). Pearson’s correlation coefficient for *GLUL* and *ASNS* expression is *r* = − 0.57. **B** Expression levels according to C1 and C2 tumour type. Expression levels by tumour type are different for *GLUL* (*p < 0.05) but not for *ASNS* (not significant). **C** *GLUL* expression values separated by a cut off value of SLR 12.0. Significant difference between high and low expression group (***p < 0.001). **D** *ASNS* expression values separated by a cut off value of SLR 6.75. Significant difference between high and low expression group (***p < 0.001). **E** Kaplan–Meier survival curves stratified according to high and low *GLUL* expression. Median overall survival time is 68 months for the low *GLUL* expression group, but not reached for the high *GLUL* expression group. Log rank test for group comparison *p* = 0.1855. **F** Kaplan–Meier survival curves stratified according to high and low *ASNS*. Median overall survival not defined for the groups. Log rank test for group comparison *p* = 0.7125.

Data of expression databases revealed that *GLUL* and *ASNS* are overexpressed in HB compared to non-tumor liver tissue. All HB except one showed a higher *GLUL* expression than the non-tumour tissue. Relative *GLUL* expression levels for HB and the non-tumor tissue were 12.4 ± 1.3 and 9.7 ± 0.4, respectively (*p* = 0.0003). All HB exhibited a higher *ASNS* expression than the non-tumor tissue (SLR 7.0 ± 1.1 and SLR 5.5 ± 0.1, *p* = 0.01). Pearson's correlation coefficient for *GLUL* and *ASNS* expression in tumor samples is *r* = − 0.57 (Fig. 1A).

To further investigate the significance of the expression levels, we examined the distribution of the expression levels and the association with patients’ survival. The scatter plots of *GLUL* and *ASNS* expression revealed a high and a low expression group separated by a cut off value of SLR 12.0 for *GLUL* and of SLR 6.75 for *ASNS* (Fig. 1C and D). The *GLUL-*high (SLR 13.2 ± 0.4) and the *GLUL*-low (SLR 10.7 ± 0.5) as well as the *ASNS*-high (SLR 7.8 ± 0.8) and the *ASNS*-low (SLR 6.1 ± 0.3) groups were statistically different (*p* < 0.001). Low *GLUL* expression was associated with a reduced median overall survival of 68 months (Fig. 1E) compared to the high expression group (median of overall survival not reached); for group comparison, the log rank test revealed a *p* = 0.1855. Median overall survival of the *ASNS* high and low expression group has not been reached (group comparison: *p* = 0.7125) (Fig. 1F).

We further assessed (Fig. 1B) if these results are in line with conclusions drawn from the molecular classification scheme of HB based on the genetic profile and differentiating tumors in type C1 and type C2, type C2 being associated with a more advanced tumor stage and poor prognosis (Cairo et al. 2008). C1-type HB exhibited a higher *GLUL* expression (SLR 12.5 ± 1.1) than the C2-type HB (SLR 11.3 ± 1.5) (*p* = 0.03). The difference in *ASNS* expression of the two groups (C1: SLR 6.8 ± 0.9 and C2: SLR 7.4 ± 1.4) was not significant (*p* = 0.26). These data indicate that *GLUL* expression is associated more than *ASNS* expression with overall survival and tumor stage in HB.

### Mutations in exon 3 of *CTNNB1* and expression of *GLUL* and *ASNS* in human HB samples

**Fig. 2:**
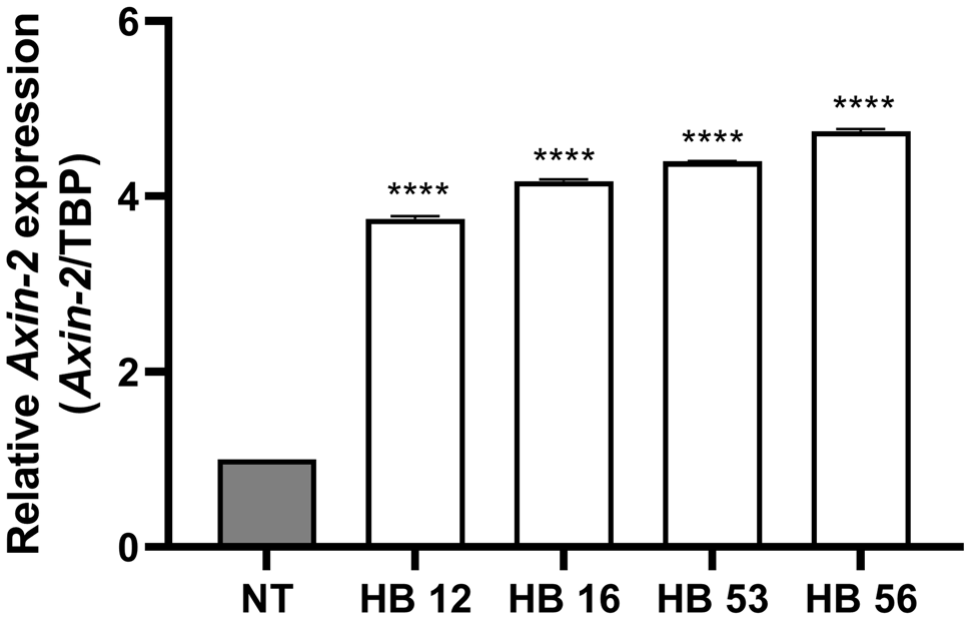
*Axin-2* expression in HB tissue samples. *Axin-2* expression was normalized to expression of *TBP*. Significant increase in tissue samples compared to normal liver tissue (NT). Data show means ± SD (*n* = 3). ****p < 0.0001 vs. NT. mRNA of HB sample 43 could not be extracted

Two of the five human HB samples, the epithelial-embryonal and one of the epithelial-fetal HBs, carried a mutation in exon 3 of *CTNNB1*. Both mutations were point mutations (Table 1). To further analyze the samples for activation of the Wnt pathway, we investigated the expression of the *Axin-2* gene, a target of β-catenin. RT-qPCR analysis of *Axin-2* expression revealed a higher expression in all HB samples compared to normal liver tissue, even in samples without *CTNNB1* mutation (Fig. 2).

**Table 1:** Hepatoblastoma samples. Histology and mutation

Tumor sample	Histology	*CTNNB1* mutation
HB12	Epithelial fetal	p.D32V, c.95A > T
HB16	Epithelial fetal (> 80% fetal)	No
HB43	Mixed (40% mesenchymal, 60% epithelial (45% fetal, 15% embryonal))	No
HB53	Epithelial embryonal	p.G34E, c.101G > A
HB56	Epithelial fetal	No

*p* protein sequence, *c* coding DNA sequence

**Fig. 3:**
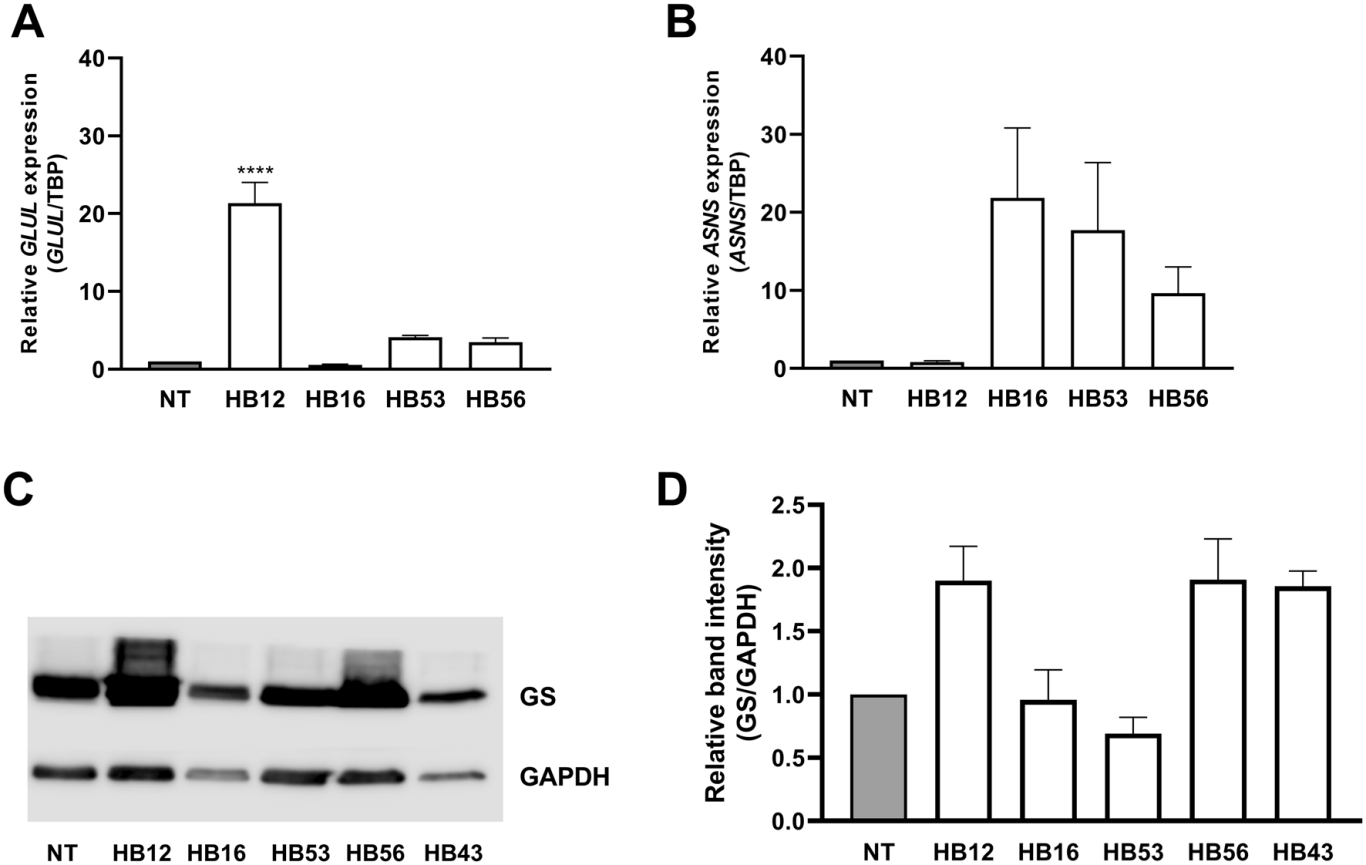
Gene expression and GS protein abundance in HB tissue samples.** A** *GLUL* expression. GS mRNA is higher than in normal tissue (NT) in all but HB16 tissue samples. mRNA of HB sample 43 could not be extracted. *GLUL* expression was normalized to expression of *TBP*. Data show means ± SD (*n* = 3). **B** *ASNS* expression. ASNS mRNA in the HB tissue samples is increased in all samples but HB12 compared to normal liver tissue. mRNA of HB sample 43 could not be extracted. *ASNS* expression was normalized to expression of *TBP*. Data show means ± SD (*n* = 3). **C** Western blot of GS. A representative experiment is shown. **D** Densitometric quantification. Relative ratio of GS/GAPDH density was normalized to ratio obtained in NT. Data show means ± SD (*n* = 5)

From the RT-qPCR analysis (Fig. 3A), all HB samples except HB16 showed an increased *GLUL* expression compared to normal liver tissue. GS levels in Western blots of tumor samples HB16 and HB53 and normal liver tissue were comparable, but higher in the other three tumor samples (Fig. 3C, D). RT-qPCR revealed an increased expression of *ASNS* for all HB samples except sample HB12 compared to normal liver tissue (Fig. 3B). Under the experimental conditions adopted for the Western blot analysis, ASNS was not detectable in normal liver tissue or HB samples.

### Embryonal hepatoblastoma cell cultures—*GLUL* and *ASNS* expression, corresponding protein levels and effect of ASNase and MSO

To investigate specifically embryonal HB cell lines, we investigated the expression of *GLUL* and *ASNS* in the HB cell lines Huh-6 and HepT1. We also assessed the effect of glutamine depletion due to the GS inhibitor MSO and ASNase, which hydrolyses Asn and Gln, on *GLUL* and *ASNS* expression, levels of the two proteins and cell viability.

**Fig. 4:**
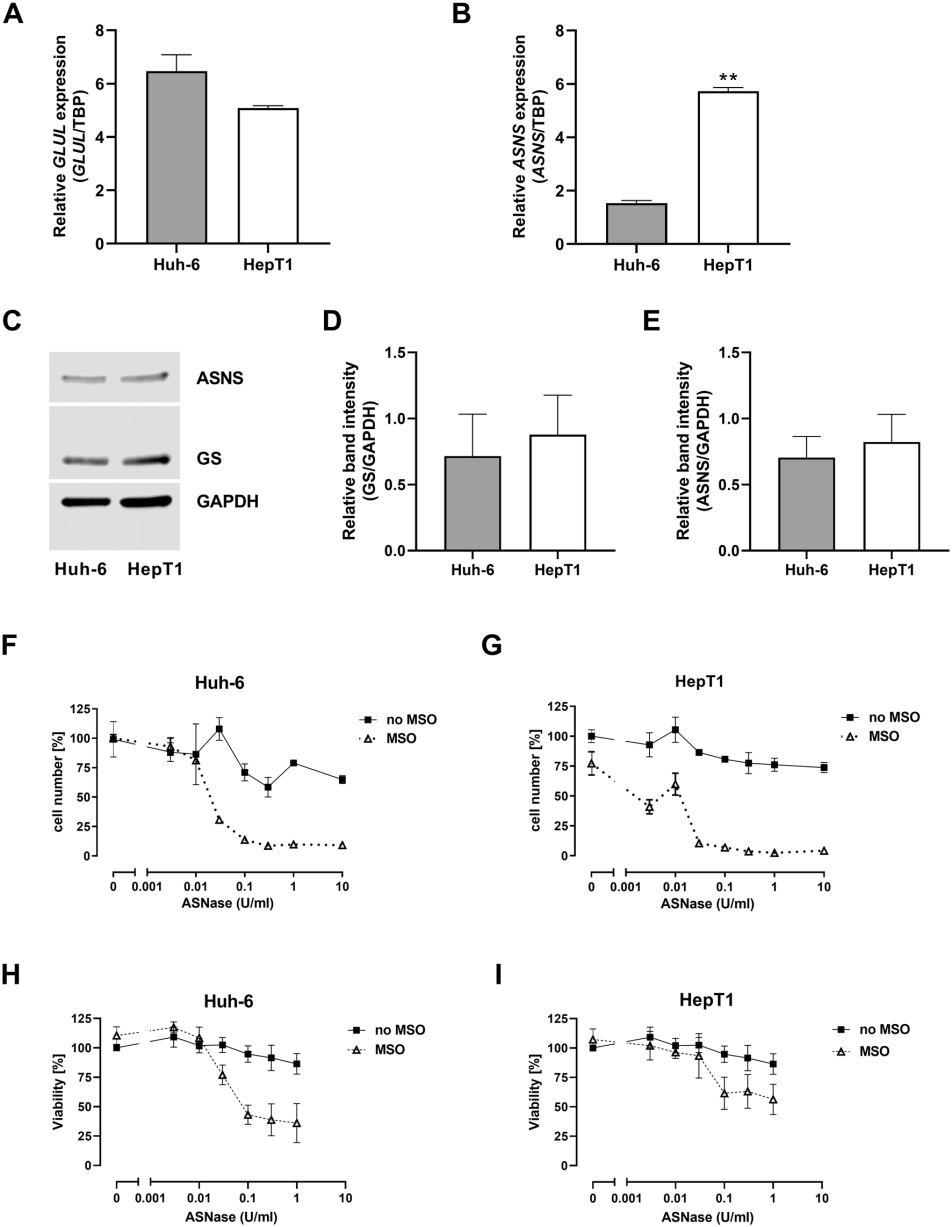
Gene expression, protein abundance and effect of glutamine depletion in HB cell lines. **A** *GLUL* expression. GS mRNA levels in Huh-6 and HepT1 cells are comparable. *GLUL* expression was normalized to expression of TBP. Data show means ± SD (*n* = 3). **B** *ASNS* expression. Significantly higher ASNS mRNA level in HepT1 cells (**p < 0.01). *ASNS* expression was normalized to expression of TBP. Data show means ± SD (*n* = 3). **C** Western blot of GS and ASNS. A representative experiment is shown. **d** Densitometric quantification of GS band. Data show means ± SD (*n* = 5). **e** Densitometric quantification of ASNS band. Data show means ± SD (*n* = 5). GS and ASNS protein abundances in Huh-6 and in HepT1 cells is comparable. **f** Effect of glutamine depletion on cell number in Huh-6 cells. **G** Effect of glutamine depletion on cell number in HepT1 cells. **H** Effect of glutamine depletion on cell viability in Huh-6 cells. **I** Effect of glutamine depletion on cell viability in HepT1 cells. In each experiment, treatment with the combination showed a greater effect than the treatment with the single substances. Results are expressed as per cent [%] of untreated control cultures. Data show means ± SD (*n* = 3). The logarithmic plot of the *X*-axis is modified to show the values without ASNase. If MSO was used, the concentration was 1 mM

Huh-6 and HepT1 cells exhibited comparable expression levels of *GLUL* (Fig. 4A), but *ASNS* was four-fold more expressed in HepT1 than in Huh-6 cells (*p* < 0.01, Fig. 4B). At protein level, GS and ASNS were clearly detectable in both cell lines and showed comparable expression levels (Fig. 4C–E).

Treatment with ASNase alone caused a slight decrease of cell number (Fig. 4F and G) and cell viability (Fig. 4H and I) in both hepatoblastoma cell lines. ASNase combined with 1 mM MSO reduced the cell number by more than 90% in both cell lines (Fig. 4F and G), and the cell viability by around 70% in Huh-6 cells (Fig. 4H) and by around 40% in the HepT1 cells (Fig. 4I). The EC50 for the dose–response curves of the combination therapy was in the range of 0.01–0.1 U/ml ASNase with 1 mM MSO. MSO alone had no effect on cell number and viability of HuH6 cells (Fig. 4F and H). In HepT1 cells, MSO did not impair cell viability (Fig. 4I) but reduced cell number by about 25% (Fig. 4G). Changes in cell viability (Fig. 4H and I) determined by the MTT assay are in line with the results of the cell count investigation and show strong inhibition by the combination treatment but not by treatment with the single substances. The small effect of MSO on cell number of HepT1 cells is not seen in Huh-6 cells.

**Fig. 5:**
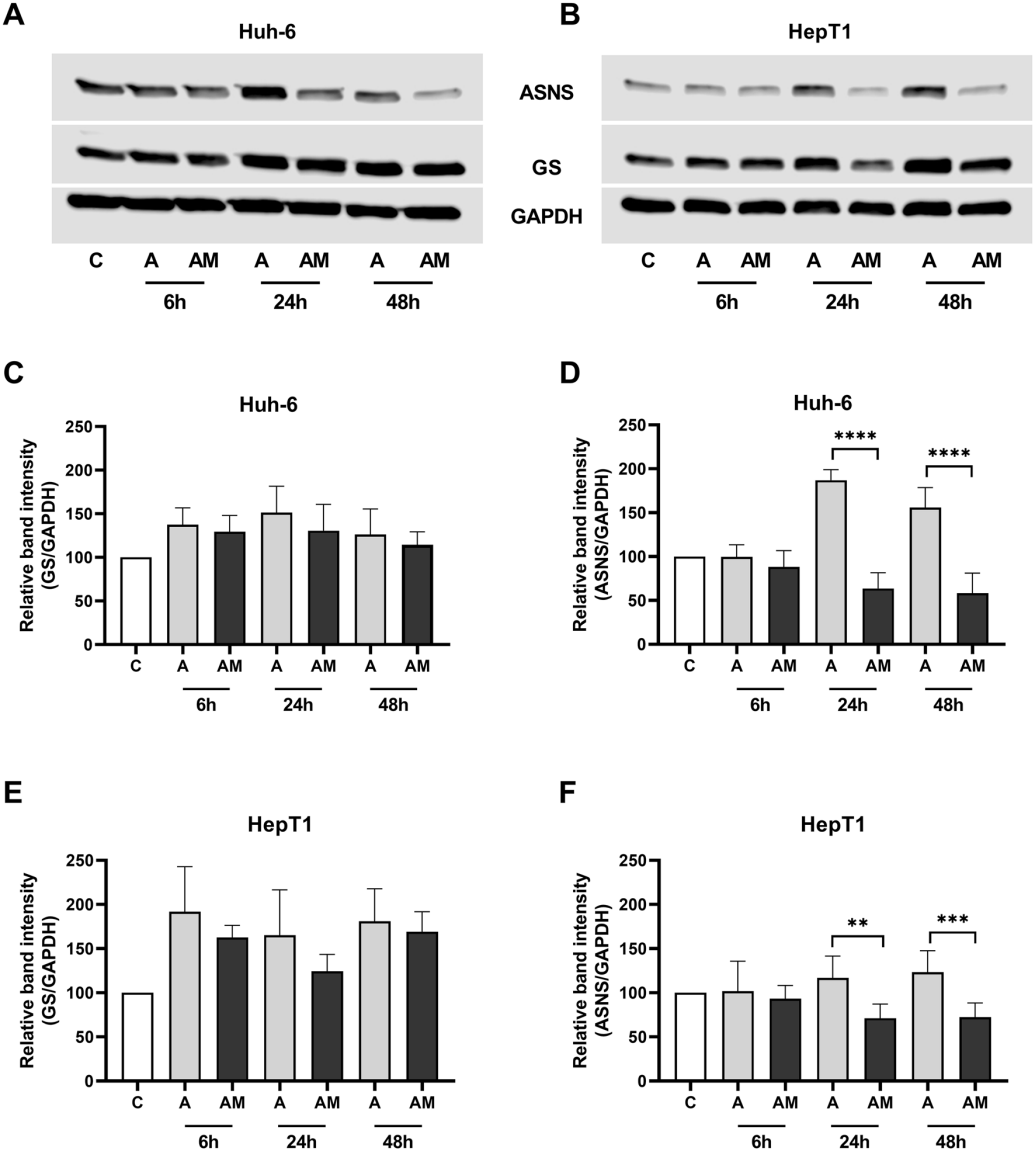
Effect of ASNase (A) and ASNase + MSO (AM) on GS and ASNS protein abundance in HB cell lines. **A** Western blot of GS and ASNS in Huh-6 cells and **B** in HepT1 cells. Representative experiments are shown. **C** Densitometric quantification of GS band in Huh-6 cells and **E** in HepT1 cells. ASNase increases GS protein abundance more than ASNase + MSO. **D** Densitometric quantification of ASNS band in Huh-6 cells and **f** in HepT1 cells. ASNS protein abundance is increased by ASNase, but reduced by ASNase + MSO. Relative ratio of GS/GAPDH density and of ASNS/GAPDH density were normalized to ratio obtained in NT. Data show means ± SD (*n* = 5). **p < 0.01; ***p < 0.001; ****p < 0.0001

Treatment with ASNase alone increased GS protein in both cell lines more than the treatment with a combination of ASNase and MSO (Fig. 5A–C, E). In both cell lines, ASNS protein was also increased after ASNase treatment alone, whereas the combination treatment led to a decrease of ASNS protein (Fig. 5A, B, D, E).

**Fig. 6:**
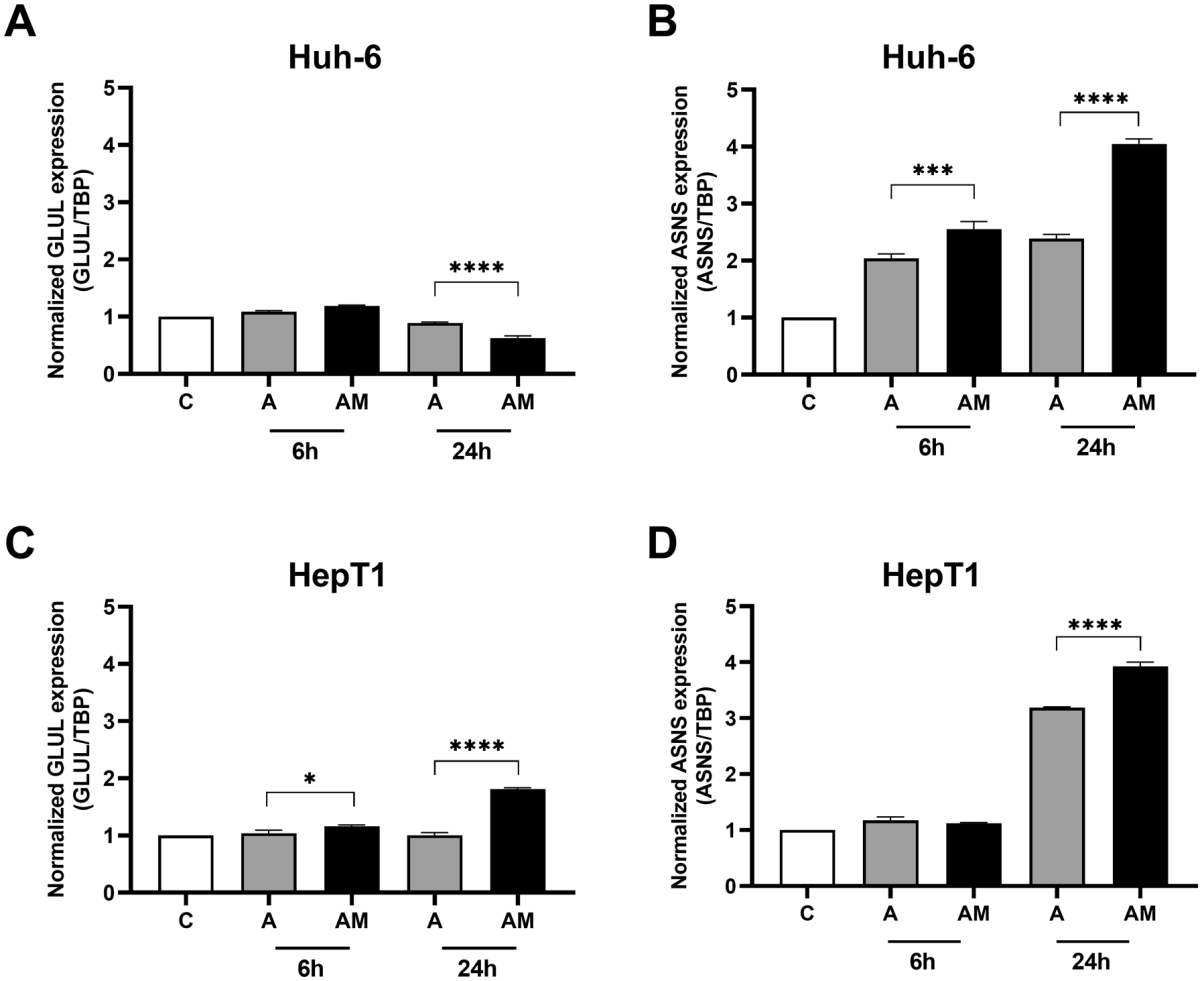
Effect of ASNase (A) and ASNase + MSO (AM) on *ASNS* and *GLUL* expression in HB cell lines. **A** *GLUL* expression in Huh-6 cells and **C i**n HepT1cells. GS mRNA is decreased in Huh-6 cells, but increased in HepT1 cells by ASNase + MSO. **B** *ASNS* expression in Huh-6 cells and **D** in HepT1 cells. ASNS mRNA in both cell lines is increased more by the combination than by ASNase alone. Relative *GLUL* expression (*GLUL* /TBP) and relative *ASNS* expression (*ASNS* /TBP) were normalized to expression in controls. Data show means ± SD (*n* = 3). *p < 0.05; ***p < 0.001; ****p < 0.0001

Treatment of the cell lines with ASNase had little effect on GS mRNA (Fig. 6A, C). Its combination with MSO led to a small decline of GS mRNA in Huh-6 cells (Fig. 6A) and an increase in HepT1 cells (Fig. 6C). Both treatments led to an increase in ASNS mRNA concentration in both cell lines, which was more pronounced with the combination treatment (Fig. 6B, D).

## Discussion

The analysis of microarray data points to a higher *GLUL* expression compared to normal liver tissue in all but one hepatoblastoma samples. Since activation of the Wnt/β-catenin signaling pathway promotes *GLUL* expression, high expression is an expected consequence of the high prevalence of activating mutations of components of this pathway in HB.

Stratification into high and low *GLUL* expression indicates a higher median overall survival time for patients with high expression of the enzyme. Cairo et al., who collected the data via microarray, distinguished a C1 versus a C2 type HB on the basis of a gene signature comprising 16 genes with statistically significant differences in 2-year overall survival rates of 92% and 44% respectively (*p* < 0.001). Interestingly, although C1 and C2 HB significantly differ for *GLUL* expression (Fig. 1B), the C1 group also includes 12.5% tumors with low *GLUL* expression, and vice versa, the C2 group includes 37.5% hepatoblastomas with high *GLUL* expression. The activated Wnt pathway causes high expression of *GLUL* with low variation*. ASNS* expression in normal cells is primarily regulated by the Amino Acid Response (AAR), and the Unfolded Protein Response (UPR) pathway. For tumors, an influence by other factors such as p53 has also been described (Chiu et al. 2019). The lack of a corresponding activation of *ASNS* expression as present for *GLUL* expression that overrides possible regulatory mechanisms for expression explains the lower expression of *ASNS* and its larger variation.

The tumorigenesis of hepatoblastoma can explain the higher median overall survival time in high *GLUL* expressing patients, while in fact a worse prognosis could be expected given the positive effects of Gln on cell viability and proliferation. An embryonic cancer stem cell, or a multipotent stem cell with the potential to develop either to an epithelial or to a mesenchymal direction, is considered the original cell of hepatoblastoma. Malignant transformation at different stages of development or arrest of a transformed cell at a certain stage of differentiation lead to the formation of tumor subtypes. The embryonal and fetal tumor subtypes have gene profiles similar to the corresponding developmental stages of hepatogenesis. The fetal type is more differentiated, as indicated by the higher *GLUL* expression and GS abundance, and is endowed with the most favorable prognosis (Armengol et al. 2011; Cairo et al. 2008; Lopez-Terrada et al. 2009b; Sumazin et al. 2017). The favorable prognosis of the fetal tumor type is, therefore, consistent with the overall longer survival observed in the high *GLUL* group. Studies on the prognostic significance of gene signatures define different gene profiles, but they all have in common the overrepresentation of the fetal type in the prognostically more favorable group (Adesina, 2009; Cairo et al. 2008; Sumazin et al. 2017).

The hepatoblastoma panel examined here was very heterogeneous, consisting of different tumor subgroups. All samples showed *GLUL* expression at both mRNA and protein level. A mutation in the *CTNNB1* gene was found only in two samples. Investigations in different cell lines and tumor types have shown that these mutations affect the ubiquitination recognition motif of β-catenin leading to its decreased degradation and nuclear accumulation and in this way to activation of the Wnt pathway (Al-Fageeh et al. 2004; Lasota et al. 2015; Terris, 1999). The mutations were found in a variety of tumors such as hepatocellular carcinoma, central nervous system tumors, pancreatic and endometrial tumors (COSMIC 2021; Kim and Jeong 2019)**.** A modest correlation between *CTNNB1* gene mutations and *GLUL* expression at the protein level determined by GS staining was also found in samples of hepatocellular adenoma and carcinoma (Hale, 2016).

Since mutations in genes other than *CTNNB1* can also lead to an activation of the Wnt/β-catenin signaling pathway, it cannot be excluded that an activated signaling pathway was present in the other samples. Therefore, we analyzed the HB samples for expression of *Axin-2,* another target gene of the Wnt/β-catenin signaling pathway. All HB samples showed increased *Axin-2* expression, indicating that an activated Wnt/β-catenin signaling pathway may be present in samples without CTNNB*1* mutation.

Since a single hepatoblastoma usually consists of different subtypes in varying proportions, it is difficult to reach definite conclusions from data obtained from tissue homogenates, given that the results represent all the subtypes present in the sample. Investigations at the histological level are more appropriate for obtaining information on the tumor subtypes. In previous studies, we found that GS staining is evident in the epithelial areas, especially in those with fetal morphology, but not in areas with mesenchymal morphology (Schmidt et al. 2014). This corresponds to the results of other reports, although the frequent absence of GS staining in epithelial embryonal areas is also described (Huang et al. 2017).

Investigations on cell lines and xenotransplants can provide further information about tumor subtype-specific properties. The best studied example is the HepG2 cell line, which was originally described as a hepatocellular carcinoma cell line, but is now considered to be derived from a hepatoblastoma (Lopez-Terrada et al. 2009a). HepG2 has a large in-frame deletion in the *CTNNB1* gene comprising 116 codons of exons 3 and 4 (Koch et al. 1999) and a mutation of the TERT promoter (Cevik et al. 2015) and represents the fetal tumor subtype with high *GLUL* expression and GS abundance (Lopez-Terrada et al. 2009b). Depletion of glutamine by a combination of the inhibitor of the glutamine synthetase methionine L-sulfoximine and asparaginase, which hydrolyses also Gln (Covini, 2012), leads to a significant inhibition of cell growth in vitro and inhibition of the growth of xenograft in mice (Chiu et al. 2014; Tardito et al. 2011).

We have studied two cell lines which have different characteristics, carrying different mutations: Huh-6 cells have a missense mutation in the *CTNNB1* gene (c.101G > T) (de La Coste, 1998), which leads to activation of the Wnt/β-catenin signaling pathway, and HepT1 cells carry a deletion of 76 codons in exon 3 of the *CTNNB1* gene (Koch et al. 1999). The cell lines are assigned to the embryonal tumor subtype (Crippa et al. 2017; Pietsch et al. 1996). In both cell lines, a combination of methionine-L-sulfoximine and asparaginase inhibited cell growth more than treatment with these inhibitors alone.

Treatment with asparaginase increased GS protein levels in both cell lines (Fig. 5A–C, E), but not GS mRNA levels (Fig. 6A, C). An increase of GS protein but not GS mRNA was also observed in the fetal hepatoblastoma cell line HepG2 and in three hepatocellular carcinoma cell lines (Hep3B, Huh-7, PLC-PRF-7) treated with asparaginase (Tardito et al. 2011). GS abundance is regulated by Gln via transcriptional and post-transcriptional mechanisms (Arad et al. 1976; Crook and Tomkins 1978). In particular, a mechanism, which was analyzed in more detail, involves acetylation of GS triggered by high Gln concentration which leads to binding of the Cereblon protein followed by GS ubiquitination and degradation by the proteasome (Nguyen, 2016). In our experiments, cell lines showed higher GS abundance after exposure to ASNase than controls (Fig. 5A–C, E). The glutaminolytic effect of ASNase may have reduced the concentration of Gln and, thus, lead to a reduced degradation of GS and higher GS abundance compared to controls.

GS protein levels were less increased and GS mRNA more decreased by a combination of asparaginase and methionine-L-sulfoximine compared to ASNase-only treatment. Since the combination treatment leads to a stronger inhibition of cell growth, it can be assumed that it is also associated with a stronger impairment of cell functions including the ability to adapt to changing metabolic conditions. In both cell lines, *ASNS* transcription is induced by asparaginase and by its combination with methionine-L-sulfoximine. Provided that a functional ASNS is produced, this could explain the partial resistance to asparaginase in the absence of MSO.

It has been shown that mutations in the *CTNNB1* gene lead to differential gene expression and metabolic patterns. In particular, it has been proposed that a large deletion in exon 3, with extension to exon 4, is associated with the fetal tumor subtype (Cairo et al. 2008; Crippa et al. 2017; Lopez-Terrada et al. 2009b). However, to our knowledge, there are no analyses that assign differential gene patterns or metabolic patterns to different mutation types within the embryonal subtype. The investigations on embryonal cell lines with different mutations suggest that glutamine depletion could be effective regardless of the mutation type.

The Wnt/β-catenin pathway interacts with other signaling pathways, as shown for Hippo/YAP pathway (Min, 2019; Tao, 2014). In addition, an impact of the tumor microenvironment on glutamine metabolism has been recently proposed (Castegna and Menga 2018), suggesting that complex relationships exist in vivo that are not easily documented in vitro. The investigation of glutamine sensitivity of malignant tumors must therefore be continued on models that take into account these possible complexities.

Glutamine depletion is effectively reducing cell viability of cell lines representing the fetal and embryonal subtype of hepatoblastoma and might be a therapeutic approach for these subtypes. The higher efficacy of a combination of ASNase and MSO compared to the efficacy of the single substances indicates that therapy by inhibiting GS alone may not achieve a sufficient glutamine depletion. Concomitant reduction of Gln concentration by hydrolytic cleavage may be required. As the tumor microenvironment can contribute to the glutamine supply of tumor cells (Castegna and Menga 2018), this could be more important for tumors than for cell lines. Glutamine synthetase immunostaining of tumor specimens may be helpful to identify tumors, for whom a therapy which includes GS inhibition seems suitable.

For adult hepatocellular carcinoma, ASNS has been proposed as a prognostic factor and described as a possible therapeutic target (Zhang et al. 2013). Our findings on ASNS in hepatoblastoma do not suggest that it has a comparable significance in hepatoblastoma. The microarray data show, indeed, no dependence of overall survival on *ASNS* expression.

## Conclusion

In hepatoblastoma, high *GLUL* expression is associated with higher median overall survival, explained by the specific tumorigenesis pathway which includes the development of a more differentiated, prognostic favorable fetal subtype with high *GLUL* and GS expression. On the contrary, in contrast to hepatocellular carcinoma, asparagine synthetase is not a prognostic factor for survival in hepatoblastoma.

Since hepatoblastomas are usually composed by areas representing different subtypes with specific characteristics, it would be necessary to demonstrate the effectiveness of a therapeutic approach for each individual tumor subtype. However, our studies on cell lines carrying different mutations suggest that the comparatively malignant embryonal subtype is also responsive to glutamine depletion, as already shown for the fetal subtype. Further studies in more complex models, however, are needed to explore the efficacy of a tumor therapy targeting glutamine dependence of hepatoblastoma.
